# Role of Gut Microbiome in COVID-19: An Insight Into Pathogenesis and Therapeutic Potential

**DOI:** 10.3389/fimmu.2021.765965

**Published:** 2021-10-14

**Authors:** Ikram Hussain, Gabriel Liu Yuan Cher, Muhammad Abbas Abid, Muhammad Bilal Abid

**Affiliations:** ^1^ Department of Gastroenterology, Khoo Teck Puat Hospital, Singapore, Singapore; ^2^ Department of Hematopathology and Microbiology, Aga Khan University Hospital, Karachi, Pakistan; ^3^ Division of Infectious Diseases, Medical College of Wisconsin, Milwaukee, WI, United States; ^4^ Division of Hematology/Oncology, Medical College of Wisconsin, Milwaukee, WI, United States

**Keywords:** COVID-19, gut microbiome, cytokine release syndrome, gut–lung axis, dysbiosis, leaky gut

## Abstract

Coronavirus disease 2019 (COVID-19), caused by the severe acute respiratory syndrome coronavirus 2 (SARS-CoV-2), resulted in an unprecedented global crisis. Although primarily a respiratory illness, dysregulated immune responses may lead to multi-organ dysfunction. Prior data showed that the resident microbial communities of gastrointestinal and respiratory tracts act as modulators of local and systemic inflammatory activity (the gut–lung axis). Evolving evidence now signals an alteration in the gut microbiome, brought upon either by cytokines from the infected respiratory tract or from direct infection of the gut, or both. Dysbiosis leads to a “leaky gut”. The intestinal permeability then allows access to bacterial products and toxins into the circulatory system and further exacerbates the systemic inflammatory response. In this review, we discuss the available data related to the role of the gut microbiome in the development and progression of COVID-19. We provide mechanistic insights into early data with a focus on immunological crosstalk and the microbiome’s potential as a biomarker and therapeutic target.

## Introduction

Since it was first recognized, Coronavirus Disease 2019 (COVID-19), caused by the Severe Acute Respiratory Syndrome Coronavirus 2 (SARS-CoV-2), remains a global affliction (1). Although vaccines offer hope in curbing the pandemic (2), an improved understanding of its pathogenesis and concurrent efforts to explore preventive and therapeutic strategies remain a priority to consolidate the success of mass vaccination and herd immunity.

The clinical spectrum of COVID-19 ranges from asymptomatic to severe, life-threatening disease (3). Current understanding of pathogenesis postulates a rapid and intense hyperactivation of the immune system, resulting in critical illness and mortality (4). Older age, burden of comorbidities, obesity, immunocompromised states, malignancy or ongoing cancer treatment, and being a transplant recipient, have been strongly linked with severe, and sometimes fatal, outcomes (5–9). Evolving data suggest that a state of chronic inflammation or baseline activation of the immune system might influence the course of COVID-19 more than direct cytopathic effects of the SARS-CoV-2. Furthermore, a subgroup of patients have been noted to develop auto-inflammatory symptoms (such as Kawasaki-like disease in children and multi-system inflammatory syndrome) long after clearance of the SARS-CoV-2 virus from body, suggesting an immune dysregulation (8, 10).

In the human body, the gastrointestinal tract (GIT) is the largest immune organ (11). The pool of resident microorganisms (bacteria, viruses, and fungi) in the GIT, collectively known as the gut microbiota, not only supports mucosal immunity but also modulates the systemic immune response of the host (12). Current evidence from other respiratory illnesses indicates that the gut microbiota affects the immunity and inflammation in the lungs (13, 14). Lately, some studies have examined the association between gut microbiota and SARS-CoV-2. In this review, we present the existing data related to the intersection of gut microbiome and the host’s immune response to SARS-CoV-2. We further explore the role of gut microbiome diversity and its compositional differences as diagnostic biomarkers, and the potential of the gut microbiome as an interventional target in modifying COVID-19 outcomes.

## Significance of Gut Microbiota

The human GIT is home to about 10^4^–10^5^ bacteria per millimeter of content in the small intestine, and 10^11^ bacteria per gram of colonic content (15). In a healthy person, the gut microbiota comprises more than 100 bacterial phyla and the majority of bacteria belong to *Firmicutes, Bacteroidetes, Proteobacteria*, and *Actinobacteria*, with *Firmicutes* and *Bacteroidetes* phyla constituting over 90% of the entire gut microbiota (16). The microorganisms and their combined genetic material make up the gut microbiome, which outnumber the human genome by about 150 times (17). The proportion of the various phyla remains quasi-stable and unique for an individual, although a shift can be observed during a change in health status. For example, the gut microbiome in the elderly has been observed to drift away from *Firmicutes* and towards *Proteobacteria* and *Alistipes* (18).

The gut microbiota exists in a symbiotic relationship with its host. It facilitates the synthesis of vitamins and fermentation of carbohydrates and other undigested nutrients and aids in the delivery of essential nutrients like short-chain fatty acids (SCFAs) to colonic epithelial cells. In addition, it also regulates mucosal permeability and provides deterrence against pathogenic microbes. More importantly, the microbiota plays an indispensable role in the preservation of intestinal homeostasis by modulating local and systemic immune responses of the host (19). The microbiota protects the GIT by (a) acting as a competitor against binding of pathogenic microbes, (b) neutralizing pathogens with their anti-microbial metabolites, (c) keeping the local immune system in a perpetual vigilant state, and (d) regulating the innate and adaptive immunity.

In a healthy person, the proportion of the various phyla mostly remains quasi-stable and unique for an individual (18). An imbalanced state is described as “gut dysbiosis”, a condition characterized by an alteration in the abundance or composition of the microbiota. Gut dysbiosis may occur with aging, dietary effects, drugs, gastrointestinal infections, and anatomical alterations of the GIT. A significantly dysbiotic state may predispose to the diseases of GIT, such as *Clostridioides difficile* enterocolitis, which is associated with prolonged and recurrent broad-spectrum antibiotic usage (20). Since gut microbiota modulates the fine balance between pro- and anti-inflammatory systemic responses, a dysbiotic state has also been associated with non-gastrointestinal systemic illnesses such as malignancy (12), type 2 diabetes mellitus (21), non-alcoholic steatohepatitis (22), coronary artery disease (23), neurodegenerative diseases (24), and depression (25).

## The Gut–Lung Axis

The GI and respiratory tracts share a common mucosal immune system, known as the gut–lung axis (26, 27). From birth, both tracts receive their quota of microbiota *via* the oral route (28), and subsequently establish a differing but internally quasi-stable genre of microorganisms or microbiota (29). Although the microbiota of both tracts consists of similar phyla, they differ at the level of species in composition and density. Understandably, studies on respiratory microbiota have been complicated by tedious and invasive methods for collection of uncontaminated lower respiratory samples, and most data have been derived from mice models where lung tissue can be aseptically obtained (30). Consequently, there is growing excitement in understanding this complex immunological intersection.

Throughout the lifespan of an individual, established microbiota of both tracts contribute to the gut–lung axis, modulating both local and systemic immune responses when faced with a pathogenic threat. The axis is believed to be bidirectional, affecting the immune response of either tract when one site is activated (31). Using a germ-free murine model, Ichinohe et al. demonstrated potentially deleterious effects on respiratory immune responses after alteration of the gut microbiota with antibiotics (32). Other studies have also found that gut microbiota alterations result in abnormal activation of the immune system, predisposing to respiratory illnesses such as asthma, lung allergic responses, and chronic respiratory diseases (27, 33). Conversely, animal studies have also revealed an alteration in the gut microbiota after respiratory viral and bacterial infections (34–36). This distant effect is believed to be communicated by activation of the systemic immune system, with dysbiosis of either tract feeding into the other.

## Role of Gut Microbiota in Respiratory Virus Infection

While the immune-related interactions between resident gut and respiratory tract microbiota are yet to be explored, a conceptual understanding of the impact of the gut microbiota in patients with COVID-19 may be extrapolated by examining the existing evidence of its role in non-SARS-CoV-2 respiratory virus infections.

### Evidence From SARS-CoV-1 Infection

Many respiratory viral illnesses are commonly accompanied by GI symptoms. Previous studies during the severe acute respiratory syndrome (SARS) outbreak in 2002 showed that diarrhea was a common symptom and occurred in 16%–73% of patients. The Severe Acute Respiratory Syndrome Coronavirus (SARS-CoV-1) was not only known to infect the lung epithelial cells but also the immune cells, triggering an intense immune response with elevation in Th2 cytokines (37). It was postulated that high levels of circulating pro-inflammatory cytokines altered the gut microbiota and compromised intestinal integrity. The resultant “leaky” gut allowed translocation of bacterial products and antigens into the circulation, further exacerbating the illness (38). Due to the bidirectional nature of the gut–lung axis, an alteration in gut microbiota further augments the respiratory immune responses, conceivably resulting in a vicious perpetuation of systemic inflammatory response (32).

### Evidence From Other Community Respiratory Viruses

Using a mouse-model, Deriu et al. demonstrated that respiratory viral infection due to influenza resulted in gut dysbiosis predisposing to secondary Salmonella infection *via* circulatory type I interferons (34). Similarly, Wang et al. demonstrated indirect intestinal inflammation with influenza infection in a mouse-model occurring *via* microbiota-mediated Th17 cell-dependent inflammation (36). Several studies have reported gut dysbiosis after respiratory viral infection (39, 40). Groves et al. showed that gut dysbiosis, in the form of an increase in *Bacteroidetes* and a decrease in *Firmicutes* phyla abundance, occurred in mice models with respiratory syncytial and influenza virus infections, but not in those vaccinated with live attenuated influenza viruses (41). Furthermore, elevated levels of colonic Muc5ac and fecal lipocalin-2 in the pathogenic infection group suggest the presence of low-grade gut inflammation during respiratory virus infection.

Respiratory virus infection may also cause dysbiosis in lung microbiota, modulating both local immune responses within the lung parenchyma and systemically. Due to the difficulties in sampling of lung microbiota, only a few studies have examined the role of respiratory pathogens in altering lung microbiota. Molyneaux et al. reported an increased proportion of *Proteobacteria* and potentially pathogenic *Haemophilus influenzae* in the lower respiratory tract microbiota in rhinovirus-infected patients with chronic obstructive pulmonary disease (42). Using a mouse-model inoculated with intranasal H1N1 influenza virus, Gu et al. demonstrated a bacterial class shift in the lung microbiota, which persisted even during the recovery period (43). Notwithstanding the limitations in available studies, a common theme has emerged showing a link between respiratory virus infection and an alteration in the gut and respiratory tract microbiota, with the presence of inflammation of the GIT.

## Role of Gut Microbiota in the Pathogenesis of COVID-19

**Figure 1:**
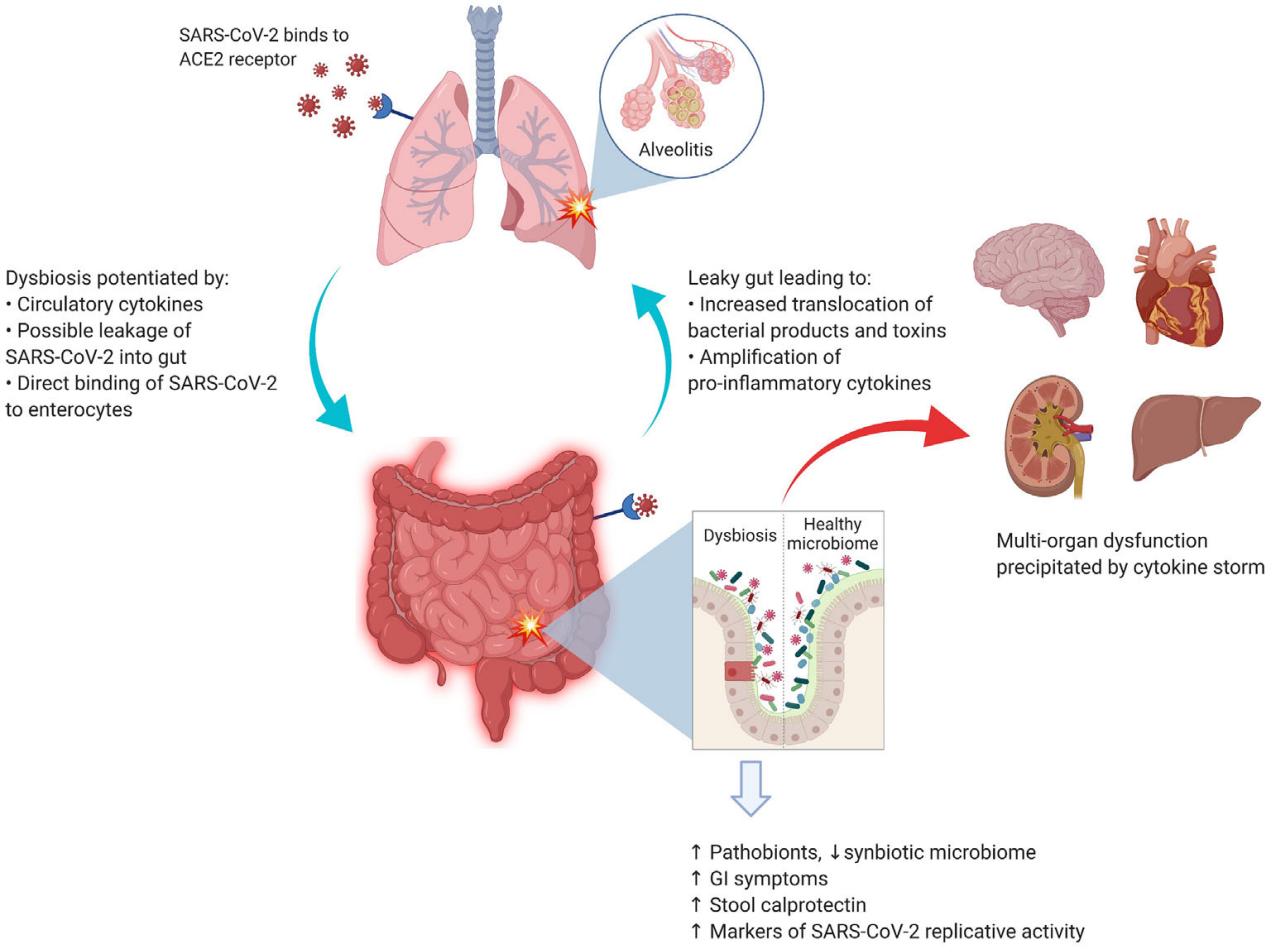
SARS-CoV-2 and the lung–gut axis: SARS-CoV-2 virus enters the alveolar cells by binding with ACE2 receptors, which are also abundant on the surface of enterocytes. The implication of direct infection of enterocytes by SARS-CoV-2 is still being explored. The circulatory cytokines from alveolitis (and/or direct viral infection of the enterocytes) cause the GI dysbiosis with resultant alterations in GI mucosal barrier. The entry of bacterial products and toxins from the GIT floods the circulatory system with more pro-inflammatory cytokines. Image was created with Biorender.com.

Although no specific interaction between any gut microbial species and SARS-CoV-2 has been identified to date, there is indirect evidence (44, 45) that gut microbiota may have a role in the overall pathogenesis of COVID-19, as summarized in **Figure 1**. Taking the corollary further from non-SARS-Cov-2 virus-mediated gut dysbiosis, it is conceivable that infection with SARS-CoV-2 may also be impacted by immunological interactions with the gut microbiota.

### Pathogenesis of GI Symptoms in COVID-19

About half of patients with COVID-19 develop GI symptoms, which often precede the respiratory symptoms (45). In the respiratory tract, the SARS-CoV-2 infects the alveolar cells by binding to angiotensin-converting enzyme 2 (ACE-2) receptors. Interestingly, these receptors are also abundantly expressed on the surface of enterocytes (46), where they play an important role in maintaining the homeostasis of microbiota and mucosal inflammation (47). In one patient with COVID-19, Xiao et al. detected SARS-CoV-2 RNA, viral nucleocapsid protein, and ACE-2 in the epithelial cells of the esophagus, stomach, duodenum, and rectum (48). Several authors have also reported detection of SARS-CoV-2 RNA fragments, but not the whole virus, in stool samples (49, 50). A study by Lamers et al. also demonstrated that SARS-CoV-2 was able to infect enterocyte lineage cells in a human intestinal organoid model (51). Unfortunately, due to the scarcity of autopsy studies and pragmatic restrictions on endoscopic examinations of the GIT, there are only limited data to support this hypothesis *in vivo*. It also remains unknown how SARS-CoV-2 may survive the acidic gastric environment to directly infect enterocytes. Overall, it is unclear whether the GI symptoms of COVID-19 are a result of primary infection of the GIT or derived from other indirect mechanisms mentioned below.

### Fecal Shedding of SARS-CoV-2

SARS-CoV-2 is principally transmitted by respiratory droplets, but evidence is accumulating for fecal-oral transmission. The hypothesis is supported by the presence of GI symptoms, detection of SARS-CoV-2 nuclear fingerprints in the GI mucosa, and detection of viral fragments in fecal samples. Interestingly, the first COVID-19 patient in the United States tested positive for SARS-CoV-2 *via* stool samples (52), and subsequent studies have consistently documented shedding of viral RNA in the stool samples in COVID-19 patients (53, 54). Furthermore, viral shedding in stool samples has been observed to persist longer than that in respiratory samples (55, 56).

### Hypercytokinemia and the Leaky Gut

Elevated serum levels of pro-inflammatory markers, such as interleukin-6 (IL-6) and interleukin-10 (IL-10), are hallmarks of severe COVID-19 infection (57). These cytokines predispose to dysbiosis, which consequently alters intestinal permeability, a state known as the “leaky gut”. This enables further entry of a multitude of bacterial products and toxins, activating a pro-inflammatory cascade. In a study in 204 patients with COVID-19, Pan et al. reported that GI symptoms worsened with increasing severity of COVID-19 (58). In another study, fecal calprotectin levels (a marker of GI mucosal inflammation) were elevated in patients who had diarrhea during COVID-19 illness (59). A recent study by Prasad et al. measured several markers of gut permeability in the plasma (60). Levels of FABP2, PGN, and LPS were significantly higher among COVID-19 patients compared to healthy subjects, suggesting translocation of pro-inflammatory antigens from a leaky gut.

### Association Between Gut Dysbiosis and Systemic Inflammation

Gu et al. first presented evidence of an altered gut microbiota in COVID-19 patients by using high-throughput sequencing of 16S ribosomal RNA to compare the gut taxa of patients with COVID-19, H1N1 influenza, and healthy controls (61). Compared to healthy controls, COVID-19 patients had significantly reduced bacterial diversity, increased abundance of opportunistic pathogens (such as *Streptococcus, Rothia, Veillonella*, and *Actinomyces*), and significantly less diverse symbiotic species. Interestingly, the altered microbial signature in COVID-19 was different from patients with the H1N1 strain.

In another study, Zuo et al. analyzed fecal samples of 15 patients with COVID-19 using shotgun metagenomic sequencing (62). The study revealed a marked increase in opportunistic pathogens and depletion of beneficial microbes as compared to healthy controls, which persisted even after clearance of SARS-CoV-2. These findings suggest an inverse correlation between gut dysbiosis and COVID-19 severity and are congruent with the conclusions of a larger multi-center study in which Yeoh et al. examined the gut microbiota in 100 patients using shotgun sequencing. Fecal samples from 27 patients were analyzed longitudinally over 30 days, showing significant dysbiosis that persisted despite clearance of SARS-CoV-2. Correlative blood samples demonstrated an association between gut dysbiosis, elevation in the inflammatory mediators, and severity of systemic inflammation (63).

Another study by Newsome et al. compared microbiota composition from stool samples of 50 COVID-19 patients with uninfected patients (64). Significant perturbations in the microbiota composition in the COVID-19 patients were observed, independent of antibiotic exposure. The gut “metabolome”, a biochemical signature derived from bacterial metabolic activity in the gut, is another method for detecting an alteration in the gut microbiota composition. In a recent study by Lv et al., fecal samples of COVID-19 patients had altered metabolomes, suggesting malnutrition and intestinal inflammation (65). These results provide new insights into the pathogenesis of COVID-19.

### Gut Dysbiosis as a Biomarker of Viral Replication

Although it was previously believed that gut dysbiosis in COVID-19 was mainly driven by inflammatory mediators from respiratory tract infection, a recent study suggests that active replication of SARS-CoV-2 in the gut may be driving the dysbiosis (66). Using *in vitro* transcriptional analysis in a SARS-CoV-2-infected cell model (with samples obtained from stools), the 3’ end of the SARS-CoV-2 genome was detected more than the 5’ end, suggesting active viral replication. Interestingly, majority of the patients had no GI symptoms, suggesting a quiescent GI infection despite active replication of SARS-CoV-2 in the GIT with dysbiosis. Moreover, on functional analysis of the gut microbiota, fecal samples with signatures of high SARS-CoV-2 burden demonstrated high *de novo* nucleotide and amino acid biosynthesis, correlating with increased bacterial proliferation. Although this was a pilot study comprising only 15 patients, further studies on alterations in the functionality of the gut microbiota may unearth the pathophysiology of COVID-19 illness.

Overall, currently expanding evidence suggests that patients with COVID-19 suffer from an alteration in the gut microbiota during and after the illness. Both systemic inflammation and replicative potential of SARS-CoV-2 in the gut may contribute towards dysbiosis.

## Clinical Implication of Gut Microbiota in COVID-19

Though limited in number, studies to date have consistently demonstrated gut dysbiosis in patients with COVID-19 (**Table 1**). A more important clinical implication lies in understanding whether, and how, gut microbiota predisposes to varying degrees of COVID-19 severity.

**Table 1:** Studies exploring the role of gut microbiota in COVID-19.

Authors	Study design	Study population	Analyses/methods	Salient findings	Limitations
**Zuo et al.** (62)	Single center, prospective	15 COVID-19 patients in Hong Kong compared against 6 subjects with community-acquired pneumonia and 15 healthy individuals	Shotgun metagenomic sequencing for profiling of GI microbiota	Significant alterations in GI microbiota (dysbiosis) in COVID-19 patients Persistent dysbiosis despite clearance of SARS-CoV-2 Positive correlation between dysbiosis and severity of COVID-19	Small sample size. Only hospitalized patients with moderate to severe COVID-19. >50% patients with COVID-19 had received antibiotics.
**Gu et al.** (61)	Single-center, cross-sectional	30 COVID-19 patients compared against 24 H1N1 patients and 30 matched healthy controls	16S ribosomal RNA gene sequencing for profiling of GI microbiota	Significantly reduced bacterial diversity (dysbiosis) with COVID-19, a significantly higher relative abundance of opportunistic pathogens, and a lower relative abundance of beneficial symbionts. Patients with H1N1 displayed lower diversity and different overall microbial composition compared with COVID-19 patients.	Small sample size. Healthy controls matched for age, sex, and BMI but not for diet and lifestyle factors. H1N1 cohort had been hospitalized for severe illness, compared to COVID-19 cohort which had disease severity classified as “general” and “severe”.
**Yeoh et al.** (63)	Prospective cohort study from two centers	100 COVID-19 compared against healthy controls	Shotgun sequencing of stool DNA for profiling of GI microbiota. Assessment of serum levels of inflammatory markers.	Significant alterations in the GI microbiota (dysbiosis) in COVID-19 patients. Dysbiosis persisted even after 30 disease post illness. Significant correlation of dysbiosis with severity of COVID-19 illness and with various serum pro-inflammatory markers.	Heterogeneous clinical management of patients. 30-day changes were studied in 27 (out of 100) patients
**Zuo et al.** (66)	Prospective cohort study from two centers	15 hospitalized patients with COVID-19	RNA shotgun metagenomics for profiling of GI microbiota. Assessment of functionality of GI microbiota and detection of replicative activity of SARS-CoV-2 virus in the GI tract.	Detection of alterations in GI microbiota (dysbiosis) with high markers of bacterial cellular building. 46.7% patients had stool positivity for SARS-CoV-2, even in the absence of GI manifestations. High replicative activity of SARS-CoV-2 in the GI tract suggesting.	Small sample size. Exact role of various microbiota profiles in determining severity of COVID-19 infection needs further studies
**Tsikala et al.** (67)	Cross-sectional (A statement across 122 countries)	42 low- or low-middle-income countries compared against 80 high- or upper-middle-income countries	Statistical analysis comparing deaths per million secondary to COVID-19 infection, against population health indicators like water current score, health efficiency, percentage rural population, proportion of diarrhoea cases secondary to inadequate sanitation and healthy life expectancy (HALE) at birth.	A statistically significant negative correlation was observed between COVID-19 mortality and populations that had a high percentage of rural residents, and a high proportion of diarrhea secondary to inadequate sanitation. As a result, a high microbial exposure to gram-negative bacteria was proposed to confer protective effects against COVID 19, possibly due to increased interferon type I levels.	Cross-sectional data based on national population health indicators. Inferential hypothesis based on effect of environmental microbiological prevalence rather than direct sequencing of human GI microbiota samples. No analysis of interferon type-I levels in study populations.
**Prasad et al.** (60)	Prospective cohort study from one center	30 hospitalized patients with COVID-19 and 16 healthy subjects.	Microbial DNA extraction and 16S rRNA sequencing in the plasma samples. Levels of gut permeability markers were also measured.	In the plasma samples of about 65% patients with COVID-19, abnormal signatures of gut microbes were seen. As compared with the healthy controls, patients with COVID-19 had significantly elevated plasma levels of gut permeability markers (such as FABP2, PGN, and LPS).	Small sample size. One-time collection of the plasma. Absence of demonstration of gut dysbiosis in the stools.
**Newsome et al.** (64)	Prospective cohort study from one center	50 hospitalized COVID-19 patients, 9 recovered patients and 34 uninfected subjects.	16S rRNA sequencing and qPCR analysis was performed on fecal DNA/RNA.	The fecal microbial composition was significantly different in the currently infected COVID-19 patients. The COVID-19 patients had increased relative abundance of *Campylobacter* and *Klebsiella*, two genera associated with GI disease. The microbiota composition was similar between recovered and uninfected patients.	Small sample size. Cross-sectional sampling.
**Lv et al.** (65)	Prospective cohort study from one center	56 hospitalized COVID-19 patients and 47 age- and sex-matched healthy subjects.	Stool samples were analyzed for various microbial biochemical products (or metabolome) using gas chromatography–mass spectrometry.	Differences in the metabolomes of COVID-19 patients were observed compared with healthy controls.	Small sample size. Absence of demonstration of gut dysbiosis in the stools. No control for diet and anti-microbial agents.

### Potential Role of Gut Microbiota in Asymptomatic/Subclinical and Mild COVID-19

As previously mentioned, clinical spectrum of COVID-19 ranges from asymptomatic to severe, life-threatening disease (3). A recent systematic review demonstrated that about one-third of patients remain clinically asymptomatic after infection with SARS-CoV-2 (68). However, a possibility of subclinical inflammatory process remains. In a systematic review involving 231 asymptomatic COVID-19 patients, almost two-thirds (63%) had inflammatory changes in the lungs on computed tomography (CT) scan (67). Irrespective of subtle inflammatory changes, a subset of patients may not mount the intense inflammatory response that portends severe illness. As this heterogeneity in clinical severity is less likely due to the existence of less virulent strains of SARS-CoV-2, or the protection from adaptive immunity given the novel nature of the virus, the immune response of the host remains the most probable factor in determining disease severity. It is unclear if any specific pattern of gut microbiota protects individuals from mounting a severe inflammatory state when infected with SARS-CoV-2. Kumar et al. highlighted a potential link between the environmental microbiota of a population and the burden of COVID-19. With data from 122 diverse countries, lower COVID-19-associated mortality was observed in countries with a higher percentage of rural population (alluding to higher gut microbial diversity), higher proportion of population residing in slums, and a lower water quality and sanitation score (69). While such observational data can be prone to confounders, these results offer some insight into the potential role of gut microbiota on the disease burden of COVID-19.

### Gut Microbiota in Severe COVID-19

There is mounting evidence that being elderly and having a chronic inflammatory state (from chronic medical conditions) predisposes to a pro-dysbiotic state (70, 71). It is unlikely a coincidence that the highest rates of morbidity and mortality from COVID-19 have also been observed in the elderly, those with underlying chronic medical conditions, and among immunosuppressed patients with cancers (5–9). COVID-19 disease severity is likely host dependent and driven by the inflammatory response. In autopsy samples from a patient with severe COVID-19, inflammatory cells were observed in the lungs (72), suggesting an intense inflammatory response (8). Furthermore, studies have also reported elevated plasma levels of pro-inflammatory cytokines such as interleukin-1 (IL-1), IL-6, and tumor necrosis factor alpha (57, 73), in severe COVID-19. In patients prone to gut dysbiosis, further inflammatory triggers may tip the balance over to a leaky gut, resulting in a self-perpetuating inflammatory feedback circle. Notably, two small studies showed a direct correlation between severe COVID-19 and gut dysbiosis (62, 63) Another two studies have shown that patients with severe COVID-19 experienced more pronounced GI symptoms, along with higher levels of stool calprotectin (an indicator of GI inflammation and disrupted mucosal integrity) (58, 59). This supports the concept of an immunological crosstalk between the lungs and gut, presumably moderated by the gut microbiota (12). There is still a lack of data assessing the role of gut microbiota in a high-risk cohort (such as elderly or cancer patients) with COVID-19, although a study on the impact of probiotics on health and immunity in elderly and diabetic patients, and response to COVID-19 vaccination, is underway (**Table 3**).

## Therapeutic Potential of Gut Microbiome for COVID-19

Given the association between gut dysbiosis and COVID-19 severity, the therapeutic potential for modulation of the gut microbiome to modify disease outcomes holds promise. However, there is no microbiota-directed therapy that has demonstrated efficacy in preventing the development or progression of COVID-19 currently.

### Potential Role of Prebiotics

Plant-based fibers exert a prebiotic effect by promoting the growth of beneficial microorganisms in the gut microbiota (e.g., *Bifidobacterium* and *Lactobacillus* spp.) while decreasing the proportion of harmful species (e.g., *Clostridia*) (74). Moreover, the fermentation of soluble dietary fibers by certain bacterial species yields several beneficial metabolites, such as SCFAs, which serve to maintain colonic mucosal integrity and modulate the immune system (75). By-products of SCFAs are also absorbed into the circulatory system and have anti-inflammatory effects. In mice models, a high-fiber diet with elevated SCFA levels was protective against allergic inflammation in the lungs, while a low-fiber diet with decreased SCFA levels resulted in increased allergic airway disease (76). Interestingly, studies from other respiratory diseases have demonstrated a reduction in mortality with intake of whole grains (77, 78). Although the beneficial effects of dietary fibers are intuitive, there is currently no direct evidence that any specific amount or type of dietary fiber is beneficial in COVID-19 illness.

### Potential Role of Probiotics

Oral probiotics are live bacteria of specific species that alter the composition of gut microbiota after reaching the intestines (74). A shift to beneficial bacterial species modulates the local and systemic inflammatory balance, with several studies demonstrating a positive impact on respiratory infections and other extra-intestinal illnesses. Using a probiotic bacterium, *Lactobacillus gasseri SBT2055* in mouse models, prevention of infection with respiratory syncytial virus was demonstrated (79). In another study on 30 elderly volunteers, *Bifidobacterium lactis HN019* ingestion was shown to enhance the cellular immunity (80). Placebo controlled clinical trials with probiotics (using *Lactobacillus rhamnosus GG*, *Bacillus subtilis*, and *Enterococcus faecalis*) have also demonstrated significant improvement in patients with ventilator-associated pneumonia (81, 82).

Naturally, if dysbiosis is indeed involved in the pathogenesis of severe COVID-19, probiotics appear to be among the more convenient, efficient, and potentially safe strategies. After initial reports of gut dysbiosis in patients with severe COVID-19, the National Health Commission (of China) recommended the use of probiotics to maintain gut microbial homeostasis and prevent secondary bacterial infections (83). Given the dearth of data pertaining to SARS-CoV-2 and the relative safety of probiotics, the rapid promulgation in favor of probiotics seems reasonable while awaiting further evidence. Although there is no direct evidence yet showing the efficacy of any specific strain of probiotic against COVID-19, several registered trials are currently examining the therapeutic potential of various probiotics formulations in COVID-19 (**Table 3**).

### Potential Role of Fecal Microbiota Transplantation

FMT is a process of actively transferring colonies of fecal bacteria from a healthy person into the GIT of another individual. The process aims to restore the composition of gut microbiota back to a healthy state. As mentioned earlier, FMT is an effective therapy for recurrent or refractory *C. difficile* enterocolitis (84). Given the proposed role of gut microbiota in the abnormal activation of immune responses in the COVID-19, FMT can potentially be explored as a therapeutic strategy. A recent case report (85) described two patients with rapid resolution of COVID-19 after FMT was undertaken to treat concomitant *C. difficile* infection. However strong may be the hypothesis and surrounding speculations, FMT should not presently be recommended as a therapy against COVID-19 due to the scarcity of large-scale studies. To explore further, a clinical trial (FeMToCOVID) is currently registered at the clinicaltrials.org (NCT04824222), though it has not yet started recruiting patients.

## Unanswered Questions and Future Directions

Despite the efficient pace of clinical trials evaluating new and repurposed agents for COVID-19, success has been modest at best. Although still in nascent stages, evolving evidence signals a probable link between gut microbiota and the host’s immune response to COVID-19. However, the exact mechanism and extent of the role of gut dysbiosis in disease severity remain to be elucidated. This is further compounded by the inherent challenges associated with designing microbiome studies. Another possible angle would be to define the state of a leaky gut more clearly with biomarkers and cutoff criteria, to also enable clarification on whether patients who have developed an inflammatory cascade are still amenable to therapeutic gut microbiota modulation.

Besides exploration of any potential role in the management of COVID-19, long-term consequences of gut dysbiosis should also be explored with longitudinal follow-up (**Table 2**). A search of ongoing clinical trials at the US National Library of Medicine reveals 24 registered studies assessing microbiota-targeted therapeutic options (**Table 3**).

**Table 2:** Unanswered questions and potential research methodology.

Category	Question	Potential methodology
**Susceptibility to infection with SARS-CoV-2**	Does gut microbiota play a role in the onset of infection with SARS-CoV-2?	To characterize the diversity of gut microbiome across separate cohorts of individuals with varying risk of exposure to SARS-CoV-2.
**Onset of symptoms after infection with SARS-CoV-2**	Does gut microbiota influence development of symptoms in COVID-19?	To study differences of gut microbiome between asymptomatic and symptomatic individuals.
**Development of severe COVID-19 illness**	Does pre-existing gut microbiota predispose to different levels of severity?	To examine the baseline gut microbiota and correlate with severity of COVID-19 illness.
**Alteration in gut microbiota by the SARS-CoV-2**	Does gut microbiota get altered by the SARS-CoV-2?	To evaluate the temporal trend of gut microbiome in the COVID-19 illness.
**Persistence of gut dysbiosis after SARS-CoV-2**	Is SARS-CoV-2-induced gut dysbiosis temporary?	To examine the long-term trend in the gut dysbiosis and its associated implications.
**Alteration in intestinal permeability with SARS-COV-2**	Does SARS-CoV-2-led inflammation lead to alteration in intestinal permeability.	Detection and measurement of gut-derived bacteria and/or their bacterial products in the circulation or extra-intestinal tissues (such as mesenteric lymph nodes).
**Therapeutic and preventive roles of prebiotics and probiotics**	Do prebiotics and/or probiotics have potential to alter course of COVID-19 illness?	Assessing variety of probiotics in terms of prevention and optimization of COVID-19 illness.
**Therapeutic role of FMT in COVID-19**	Can a reset of gut dysbiosis to normal homeostasis with the FMT mitigate severe COVID-19 illness?	To assess the potential therapeutic role of the FMT in severe COVID-19 illness.

SARS-CoV-2 (severe acute respiratory syndrome coronavirus 2); COVID-19 (Coronavirus disease 2019); GI (gastrointestinal); FMT (fecal microbiota transplantation).

**Table 3:** Ongoing registered trials studying role of gut microbiota in COVID-19 (updated as of June 19, 2021).

A. Ongoing observational studies		
Trial number	Title	Summary
**NCT04669938**	Role of the Microbiota in the Evolution of the SARS-CoV-2 Disease, COVID-19, in Hospitalized Patients	Observational study looking at the effect of oropharyngeal and gut microbiota, host genotype, and immune characteristics and SARS-CoV-2 viral genome sequences on outcomes of COVID-19 infection.
**NCT04598334**	Cytokine Storm Among Bangladeshi Patients With COVID-19	Prospective study evaluating the relationship of inflammatory markers and cytokine levels in addition to gut microbiota on COVID-19 infection severity in Bangladeshi patients, at various points of illness progression.
**NCT04597736**	Relationship Between Biological Profiles and Clinical Evolutions Within the Same Cluster COVID-19 (COVIDCOLLECT)	Cohort study examining the relationship between the biological profiles observed from analysis of nasopharyngeal, saliva, blood, stool, and urine samples and the clinical evolutions within the same cluster of COVID-19 cases and their contact subjects.
**NCT04581135**	Study to Investigate Long-term Pulmonary and Extrapulmonary Effects of COVID-19	Prospective study investigating long-term pulmonary and extrapulmonary effects of COVID-19, including changes to gut microbiota.
**NCT04552340**	Epidemiologic, Clinical and Molecular Characteristics of Patients With Acute Respiratory Failure Affected by 2019-NCOV	Observational study examining factors including alveolar and nasal microbiota on predisposition to SARS-CoV2 viral infection, symptomology, treatment response, and predisposition to complications.
**NCT04497402**	Sex-Informed Data in the COVID-19 Pandemic	Observational study looking at differences in the biomarkers of different sexes during SARS-CoV-2 infection, including the gut microbiome.
**NCT04475211**	Predictors of Mortality at Day 28 of Patients Treated at Lille University Hospital for COVID-19	Retrospective observational study to evaluate predictive factors of mortality at day 28 in COVID-19 patients treated at a single center.
**NCT04451577**	Epidemiologic, Clinical, Molecular Characteristics of Hospital Employees With or Without COVID-19 Infection	Case–control study evaluating biological samples obtained from hospital employees to characterize SARS-CoV-2 pathogenesis and individual differences in susceptibility to the disease.
**NCT04410263**	Microbiota in COVID-19 Patients for Future Therapeutic and Preventive Approaches	Observational study analyzing biological samples including nasopharyngeal and alveolar microbiota to elucidate risk factors for the development of severe ARDS in SARS-CoV-2 infected patients.
**NCT04359836**	A Study to Explore the Role of Gut Flora in COVID-19 Infection	Observational study aiming to sequence and characterize the gut microbiome of COVID-19 patients during and after treatment.
**NCT04359706**	Bacterial and Fungal Microbiota of Patients With Severe Viral Pneumonia With COVID-19	Observational study comparing the respiratory and gut microbiota and inflammatory markers of critically ill COVID-19 patients with historical critically ill patients without COVID-19.
**NCT04355741**	Gut Microbiota, “Spark and Flame” of COVID-19 Disease	Observational study analyzing and comparing the gut microbiome of COVID patients across settings in the intensive care unit, hospital general ward, and self-caring at home.
**NCT04332016**	COVID-19 Biological Samples Collection	Observational collection and analysis of biological samples including gut microbiota of patients with COVID-19 infection and their caregivers.
**NCT04325919**	Coronavirus Disease 2019 (COVID-19) Study of Hospitalized Patients in Hong Kong	Observational study characterizing clinical, virological, microbiological, and immunological profiles of COVID-19 infection compared against patients hospitalized for pneumonia.
**NCT04708912**	Nasopharynx Microbiota Component and in Vitro Cytokines Production in Coronavirus Disease (COVID-19)	Observational study comparing nasopharynx microbiota composition, RNA sequences, and *in vitro* cytokine production in COVID-19 patients with mild-moderate, severe, convalescent disease and healthy controls.
**NCT04768244**	Impact of Maternal COVID-19 Disease on Breast Milk and Infant Health (MilkCorona)	Prospective study assessing the impact of maternal COVID-19 on immune, microbiological, and metabolic profile of breast milk and infant microbiota, growth, and development.
**NCT04913142**	About Oral and Gut Microbiota in Intensive Care Unit: SARS-CoV-2 (COVID-19) Infection Impact (CO-MIC)	Prospective cohort study to describe the impact of SARS-CoV-2 infection on the oral and gut microbiota of ICU patients, and to compare against the microbiota of non-COVID-19 ICU patients.
**NCT04813328**	A Pilot Study of the Effects of Helminth Infection and SARS-CoV-2 Seropositivity on Immune Response and the Intestinal Microbiota in India	Cross-sectional study to characterize the immune response and intestinal microbiota in people with and without SARS-CoV-2 antibodies and helminth infection.
**B. Ongoing studies utilizing microbiota as therapeutic target**		
**Trial number**	Title	Summary
**NCT04666116**	Changes in Viral Load in COVID-19 After Probiotics	Randomized clinical trial evaluating the capacity of a novel nutritional supplement intervention in decreasing SARS-CoV-2 viral load by nasopharyngeal swab
**NCT04621071**	Efficacy of Probiotics in Reducing Duration and Symptoms of COVID-19	Randomized controlled trial to evaluate the efficacy of probiotics in reducing the duration and symptoms of COVID-19 in a symptomatic population tested positive to SARS-CoV-2, self-caring at home
**NCT04581018**	An Evaluation of a Synbiotic Formula for Patients With COVID-19 Infection	Non-randomized clinical trial examining effect of a synbiotic health supplement on COVID-19 symptoms
**NCT04517422**	Efficacy of *L. Plantarum* and *P. acidilactici* in Adults With SARS-CoV-2 and COVID-19	Randomized controlled trial to study ability of a probiotic to reduce progression of mild COVID-19 infection to moderate/severe disease and other prognostic factors, including gastrointestinal symptoms and gut microbiome composition
**NCT04486482**	A Clinical Study to Assess the Physiologic Effects of KB109 in Patients With COVID-19 on Gut Microbiota Structure and Function	Randomized trial evaluating effects of a novel glycan on the gut microbiota of outpatients with COVID-19.
**NCT04479202**	The Effect of Berberine on Intestinal Function and Inflammatory Mediators in Severe Patients With COVID-19	Double-blinded randomized trial analyzing the effect of berberine on gut microbiota, gastrointestinal manifestations, and inflammatory markets in patients with severe COVID-19.
**NCT04420676**	Synbiotic Therapy of Gastrointestinal Symptoms During COVID-19 Infection	Clinical trial comparing a probiotic mixture to placebo in alleviating gastrointestinal symptoms and altering gut microbiome in COVID-19 infection.
**NCT04399252**	Effect of *Lactobacillus* on the Microbiome of Household Contacts Exposed to COVID-19	Randomized trial assessing the effect of *Lactobacillus rhamnosus GG* on the microbiome of household contacts of COVID-19 patients.
**NCT04540406**	NBT-NM108 as an Early Treatment for Suspected or Confirmed Symptomatic COVID-19 Patients (COVGUT20)	Open-label, randomized controlled trial assessing the feasibility and effectiveness of a novel botanical-based fixed-combination drug to modulate the gut microbiota and treat early-stage suspected or confirmed symptomatic COVID-19 patients.
**NCT04390477**	Study to Evaluate the Effect of a Probiotic in COVID-19	Prospective case–control pilot study to evaluate the possible effect of a probiotic mixture in the improvement of symptoms, the reduction in the number of days of hospitalization and the increase in the percentage of patients with negative PCR after infection with the coronavirus SARS-CoV-2
**NCT04366089**	Oxygen-Ozone as Adjuvant Treatment in Early Control of COVID-19 Progression and Modulation of the Gut Microbial Flora (PROBIOZOVID)	Randomized trial evaluating the adjuvant use of oxygen ozone therapy plus probiotic supplementation in addition to standard of care in the early control of disease progression in patients with COVID-19.
**NCT04877704**	Symprove (Probiotic) as an add-on to COVID-19 Management	Randomized trial studying outcomes in COVID-19 patients treated with adjunctive probiotics for 3 months. A sub-study, subject to participant consent, will also collect biological samples for comparative analysis.
**NCT04734886**	The Effect of Probiotic Supplementation on SARS-CoV-2 Antibody Response After COVID-19	Randomized trial assessing the impact of *L. reuteri DSM 17938* on SARS-CoV-2-specific antibody response upon and after infection in healthy adults, in addition to inflammatory markers, symptom severity and duration.
**NCT04390477**	The Intestinal Microbiota as a Therapeutic Target in Hospitalized Patients With COVID-19 Infection	Pilot study on the effect of a probiotic mixture in the improvement of symptoms, the reduction in the number of days of hospitalization and the increase in the percentage of patients with negative PCR after infection with SARS-CoV-2.
**NCT04366180**	Multicentric Study to Assess the Effect of Consumption of Lactobacillus Coryniformis K8 on Healthcare Personnel Exposed to COVID-19	Randomized controlled trial evaluating the effects of *Lactobacillus coryniformis K8* consumption on the incidence and severity of COVID-19 in health workers exposed to the SARS-CoV-2 virus.
**NCT04907877**	Role of Nutritional Support With Probiotics in Adult Outpatients With Symptomatic COVID-19: a Randomized Dietary Study	Randomized controlled trial comparing the effect of a probiotic mixture of bifido- and lactobacteria on COVID-19 severity and subsequent immune response.
**NCT04854941**	Efficacy of Probiotics (Lactobacillus Rhamnosus, Bifidobacterium Bifidum, Bifidobacterium Longum Subsp. Infantis and Bifidobacterium Longum) in the Treatment of Hospitalised Patients With Novel Coronavirus Infection	Randomized controlled open-label study analyzing the effect of a probiotic mixture consisting of *L. rhamnosus*, *B. bifidum*, *B, longum* subsp. *infantis* and *B. longum* on SARS-Cov-2 infection outcomes.
**NCT04798677**	Efficacy and Tolerability of ABBC1 in Volunteers Receiving the Influenza or Covid-19 Vaccine	Randomized, single-center trial comparing the immune response of volunteers who had nutritional supplementation with a probiotic formulation while receiving the influenza or COVID-19 vaccine.
**NCT04847349**	Live Microbials to Boost Anti-SARS-CoV-2 Immunity Clinical Trial (Live BASIC Trial)	Pilot study on the efficacy of a combination of live probiotics on antibody titers, symptom improvement and reinfection risk in unvaccinated persons previously infected with SARS-CoV-2.
**NCT04756466**	Effect of the Consumption of a Lactobacillus Strain on the Incidence of Covid-19 in the Elderly	Multi-center randomized controlled trial evaluating the effect of a probiotic strain on the incidence and severity of COVID-19, as well as the immune response to COVID-19 vaccination, in an elderly population living in a nursing home.
**NCT04922918**	Administration of *Ligilactobacillus salivarius MP101* in an Elderly Nursing Home During the COVID Pandemics	Clinical trial investigating the effect of *Ligilactobacillus salivarius MP101* on the functional, cognitive, and nutritional status, as well as nasal and fecal inflammatory profiles of elderly people living in a nursing home highly affected by COVID-19.
**NCT04793997**	Covid-19 Primary Care Support With Microbiome Therapy	Randomized controlled trial evaluating the efficacy of a probiotic throat spray in reducing symptoms COVID-19 in patients with mild to moderate symptoms. Secondary outcome of whether the spray can prevent transmission of the SARS-CoV-2 virus to household members.
**NCT04884776**	Modulation of Gut Microbiota to Enhance Health and Immunity of Vulnerable Individuals During COVID-19 Pandemic	Randomized controlled trial studying a probiotic formula in enhancing immunity and reducing hospitalization in elderly and diabetic patients.
**NCT04824222**	The Impact of Fecal Microbiota Transplantation as an Immunomodulation on the Risk Reduction of COVID-19 Disease Progression With Escalating Cytokine Storm and Inflammatory Parameters (FeMToCOVID)	Randomized phase II (open-label) and phase III (double-blinded) trial evaluating the effect of fecal microbiota transplantation (FMT) as an immunomodulator in addition to standard therapy on the risk reduction of disease progression in COVID-19 with escalating cytokine storm and inflammatory markers.

Extracted from the database of clinicaltrials.org on June 19, 2021.

## Conclusion

The role of resident gut microbiota in other respiratory illnesses has been well recognized. Furthermore, the brunt of unfavorable COVID-19 outcomes has been on elderly patients and those with chronic medical diseases, both scenarios known to have senescence-driven gut dysbiosis. Increasingly, evidence is mounting for gut dysbiosis as a predisposing factor for severe COVID-19, through a leaky gut phenomenon and resultant spillage of bacterial products and toxins. Evidence is emerging that the degree of dysbiosis correlates with the severity of COVID-19 illness. This behooves us to explore potentially preventive and therapeutic targets, such as dietary intervention and probiotics. Several ongoing trials are evaluating various pathogenic routes and therapeutic approaches. While efforts for direct anti-viral agents and vaccines are of prime significance, the gut–lung axis could still hold therapeutic potential.

## Author Contributions

IH and MBA: study concept. IH and MBA: literature search. IH, GY, and MBA: manuscript writing. MAA: critical revisions and intellectual content. MBA: supervision. All authors contributed to the article and approved the submitted version.

## Conflict of Interest

The authors declare that the research was conducted in the absence of any commercial or financial relationships that could be construed as a potential conflict of interest.

## Publisher’s Note

All claims expressed in this article are solely those of the authors and do not necessarily represent those of their affiliated organizations, or those of the publisher, the editors and the reviewers. Any product that may be evaluated in this article, or claim that may be made by its manufacturer, is not guaranteed or endorsed by the publisher.
